# Health impact and cost-effectiveness of a domestically-produced rotavirus vaccine in India: A model based analysis

**DOI:** 10.1371/journal.pone.0187446

**Published:** 2017-11-03

**Authors:** Johnie Rose, Laura Homa, Sharon B. Meropol, Sara M. Debanne, Roger Bielefeld, Claudia Hoyen, Mendel E. Singer

**Affiliations:** 1 Center for Community Health integration, Case Western Reserve University School of Medicine, Cleveland, OH, United States of America; 2 Department of Family Medicine and Community Health, Case Western Reserve University School of Medicine, Cleveland, OH, United States of America; 3 Department of Pediatrics, Rainbow Babies and Children's Hospital and Case Western Reserve University School of Medicine, Cleveland, OH, United States of America; 4 The Center for Child Health and Policy, Rainbow Babies and Children's Hospital, Cleveland, OH, United States of America; 5 Department of Population and Quantitative Health Sciences, Case Western Reserve University School of Medicine, Cleveland, OH, United States of America; 6 Research Computing, Case Western Reserve University, Cleveland, OH, United States of America; University of Liverpool, UNITED KINGDOM

## Abstract

**Background:**

Currently, Indian officials are incorporating a domestically manufactured rotavirus vaccine (based on the 116E rotavirus strain) into the country’s universal immunization program; this vaccine will cost significantly less than western rotavirus vaccines. Here, we examine the public health impact, cost, and cost-effectiveness of universal vaccination in India using the 116E vaccine. This work will allow comparison of universal 116E vaccination with other approaches to child mortality reduction, shed light on the future burden of rotavirus disease in India, and help stakeholders understand future resource needs.

**Methods:**

Using information from published literature, we developed a dynamic simulation model of rotavirus transmission, natural history, and related utilization among Indian infants followed until age five. Infection risk depended on the degree of viral shedding in the population. Infection risk and severity were influenced by age, number of previous infections, and vaccination history. Probabilities of inpatient and outpatient health services utilization depended on symptom severity. With the model, we compared a strategy of nationwide 116E vaccination to one of no vaccination. Costs were considered from the perspective of all payers (including families) and from the societal perspective.

**Results:**

We estimated that an established 116E vaccination program would reduce symptomatic rotavirus infection by 13.0%, while reducing population-wide rotavirus mortality by 34.6% (over 34,000 lives annually). Rotavirus outpatient visits would decline by 21.3%, and hospitalization would decline by 28.1%. The cost per disability-adjusted life year (DALY) averted was estimated at 3,429 Rupees (approximately $56). Predicted mortality reduction in children born during the first five years of vaccination implementation was nearly identical to that in children born in later years (34.4% versus 34.6%).

**Conclusions:**

116E vaccination of Indian infants would likely substantially reduce rotavirus-related morbidity, mortality, and utilization at a cost considered highly cost-effective by standard criteria. Nearly the entire mortality reduction benefit of vaccination was attributable to direct protection of those vaccinated, as opposed to indirect “herd immunity” effects.

## Introduction

Each year, rotavirus gastroenteritis claims the lives of an estimated 215,000 children under five years of age; 22% of these deaths are estimated to occur in India.[1] Given the minimal impact that water and sanitation measures have had on rotavirus burden in the poorest countries, vaccination appears to represent the most promising prevention strategy against the disease.[2–4], Two vaccines developed and manufactured in western countries—a monovalent live attenuated (RotaRix^TM^, GlaxoSmithKline) and a pentavalent human-bovine reassortant vaccine (RotaTeq^TM^, Merck)—have demonstrated efficacy in a handful of middle and low income countries[5–8], though neither has been tested in India. Even with significantly discounted prices[9], the sustainability of universal immunization with these more expensive western vaccines has been called into question.[10–12]

In 1993, a naturally-attenuated human-bovine reassortant strain of rotavirus was first reported isolated from a nosocomial outbreak in an Indian neonatal ward.[13] Through support from a number of international partners and donors, the strain—116E—was used to develop a monovalent oral vaccine (RotaVac^TM^, Bharat Biotechnic International). Following a recent trial showing the vaccine to be effective and well tolerated among 4,532 Indian infants, with efficacy against severe disease of 53.6% (95% CI 35.0% to 66.9%)[14], the 116E vaccine was licensed for use in India in 2014. At an estimated cost of under $1 per dose[15], this vaccine may prove a more sustainable alternative to the western vaccines. In July 2014, India’s Prime Minister announced that the 116E vaccine would be added to India’s Universal Immunization Programme (UIP)[16], giving many stakeholders reason to be optimistic about the prospects of reducing the rotavirus burden in that country. Given incomplete vaccine coverage and the modest efficacy of the Indian vaccine, though, the questions of how effective a universal rotavirus vaccination effort will be at the population level and how costly and cost-effective such a program will likely be remain unanswered. As the rollout of the new vaccine in India’s UIP begins[17], accurately forecasting the answers to these questions will allow public health decision makers in the nonprofit and government sectors to compare universal rotavirus vaccination with other approaches to child mortality reduction, to understand the likely future burden of rotavirus disease in India, and to anticipate future resource needs.

Recently published models of rotavirus vaccination with an 116E vaccine have failed to account for the role of asymptomatic infections in disease transmission and for indirect effects of vaccination on the unvaccinated (“herd immunity”).[18–20] The majority of rotavirus infections are asymptomatic, yet they appear to impart a degree of immunity comparable to that conferred by symptomatic infections.[21] In addition, fecal rotavirus shedding is common for children even after diarrhea resolves and for infected children who never develop diarrhea.[22–26] Given these facts, inclusion of asymptomatic infection is necessary for a realistic model of rotavirus transmission. Here, we describe the model we have developed and report the results of our analysis examining the health impact and cost-effectiveness of universal 116E vaccination in India.

## Methods

### The model

Using MATLAB 2012b (Natick, MA) and the hardware resources of our university’s High Performance Computing Cluster, we developed a dynamic microsimulation model of rotavirus transmission, morbidity, mortality, and utilization in India. In the base case analysis, we considered direct medical costs only (including those to any public sector entity paying for care as well as those to patients’ families, i.e. the “all-payer” perspective). In a secondary analysis, we considered costs from a societal perspective, which incorporated costs of lost productivity and costs associated with traveling for treatment. Living individuals from age 0 to 1825 days (five years) are considered *active* for the purposes of the model and are thus capable of infecting and being infected; at the end of five years, they are removed from the model. The two strategies examined were *universal 116E vaccination* at six weeks, ten weeks, and 14 weeks[27] of age (co-administered with other routine UIP vaccines) versus *no vaccination*. To represent the two different strategies, two versions or arms of the model were run in parallel, differing only in whether or not vaccination was present. **Tables 1–3** displays baseline values and ranges for parameters used in the model.

**Table 1:** Disease-related parameters—Estimated values and ranges used in sensitivity analysis.

Parameter	Value	Range	Source/Comments
Transmission constant (T)	5.1		Calibrated to match incidence rates of first infection at 6, 12, 24, 36 months as reported by Gladstone[28]
Efficacy of kth (k = 1, 2, 3) natural infection against any future infection (*γ*_*k*_)	0.39, 0.52, 0.67	Low: 0.29, 0.43, 0.59 High: 0.47, 0.59, 0.74	[28]
Decreased infectiousness of second infection	0.5	0.3, 0.7	[29, 30]
Decreased infectiousness of third, fourth infection	0.2	0, 0.4	[29, 30]
Mean duration of infection (days)	7	4, 10	[25, 31]
Probability that kth (k = 1, 2, 3, 4) infection will cause symptoms given no vaccination (*α*_*k*_*)*	0.413, 0.349, 0.235, 0.201	± 50%	[28]
Probability that symptoms of kth (k = 1, 2, 3, 4) infection will be severe[34]* if present (*β*_*k*_)	0.153, 0.221, 0.233, 0.167	± 50%	[28]
Probability of dying from severe* rotavirus given no formal treatment	0.061	0.0305, 0.1220	Calibrated to match 5-year Indian rotavirus mortality of 1/242 without vaccination[32]
Waning of protection of maternal antibodies	Protection declines linearly from 100% to 0% between birth and 13 weeks of age		[33]

* Severe infections are defined as those with a Vesikari score > = 11.

**Table 2:** Vaccine-related parameters–Estimated values and ranges used in sensitivity analysis.

Parameters	Value	Range4	Comments/Questions
Dose 1 coverage (6 weeks)	0.88	0.66, 1	[35]
Dose 2 coverage (10 weeks)	0.80	0.60, 1	[35]
Dose 3 coverage (14 weeks)	0.72	0.54, 0.9	[35]
Efficacy of vaccine against severe infection (*δ*_*sev*_)	0.536	0.35, 0.669	[15]
Efficacy of vaccine against symptomatic infection (*δ*_*sx*_)	0.346	0.216, 0.453	[15]
Efficacy of vaccine against any infection (*δ*_*any*_)	0.304	0.1988, 0.38	Based on ratio of efficacy of prior natural infection against any infection to efficacy of prior natural infection against severe infection[28]
Proportion of efficacy (*p*_*d*_) conferred by *d* = 0, 1, 2, 3 doses	0, 0.82, 0.84, 1	Low: 0, 0.62, 0.64, 1; High: 0, 1, 1, 1	[36]
Annual rate of waning of vaccine efficacy	0.086	0, 0.2	[8, 37]
Risk of intussusception	0.00003		[38]
Case fatality of intussusception	0.25		Assumption

**Table 3:** Utilization and cost parameters—Estimated values and ranges used in sensitivity analysis. (ORS = oral rehydration solution).

Parameter	Value	Range	Comments/Questions
Probability of hospitalization given			
Non-severe infection	0.0072	0.00361, 0.0108	Calculated based on [39, 40] *
Severe infection	0.0973	0.0487, 0.146	Calculated based on [39, 40] *
Probability of outpatient care given			
Non-severe infection	0.141	0.0705, 0.212	Calculated based on [39, 40] *
Severe infection	0.575	0.288, 0.863	Calculated based on [39, 40] *
Probability of access to ORS at home	0.26	0.06, 0.46	[41]
**Costs (in 2014 Rupees)**			
Cost of one dose	61	30.5, 122	[42]
Non-vaccine costs per dose (e.g. administration, transport, storage)	34	23, 45	Calculated based on [43, 44]
Hospital treatment for rotavirus			
Direct medical			
Paid by patient’s family	3937	2952.8, 4921.3	[45]
Subsidized by government	305	228.8, 381.3	[45]
Direct non-medical	64.3	48.2, 80.4	[45]
Indirect	0		[45]
Outpatient treatment for rotavirus			
Direct medical			
Paid by patient’s family	251.6	188.7, 314.5	[45]
Subsidized by government	83.9	62.9, 104.9	[45]
Direct non-medical	38	28.5, 47.5	[45]
Indirect	2.9	2.2, 3.6	[45]
Oral rehydration solution (per course)	6	5, 12	[46]
Discount rates: for costs/benefits	3%		[47]
Vaccine wastage	10%		[48–50]

* General formula: p(site | severity) = [p(severity | site) * p(site)] / p(severity)

**Fig 1:**
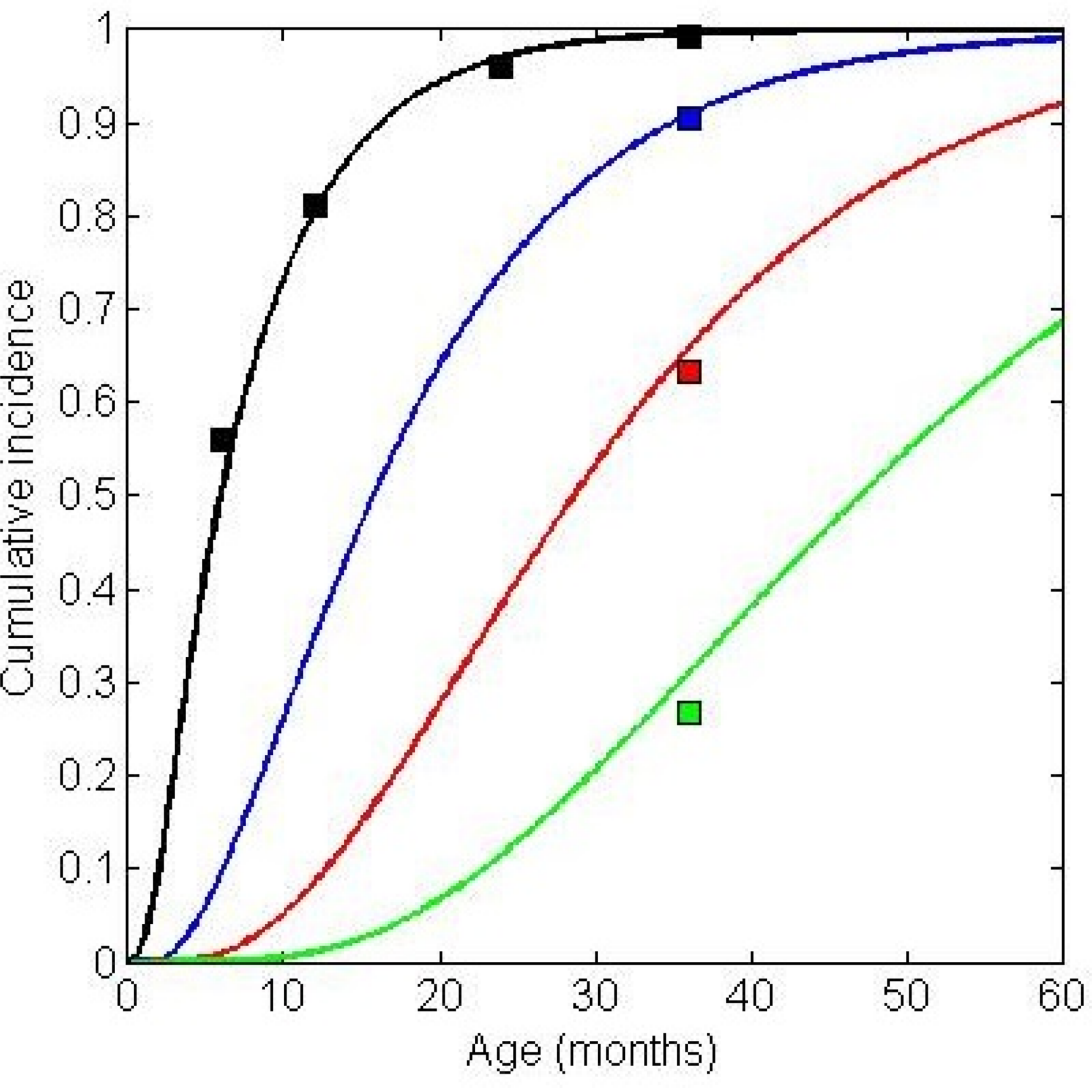
Model-generated incidence versus empirical calibration targets. Cumulative incidence of first (black), second (blue), third (red), and fourth (green) infections generated by the model without vaccination (mean time to event for 912,500 individuals—or 10 years of births at 250 births per day—each followed for five years). The colored squares represent the calibration targets as given by the Gladstone data.[28].

During each 24-hour cycle, each active individual faces a daily probability of death from non-rotavirus causes.[51] Two-hundred-fifty new individuals are born into the population of each arm daily. At birth, it is predetermined by draws from a uniform distribution whether each individual in the vaccination arm will receive all three doses, the first two doses, dose one only, or no doses of the vaccine based on actual coverage rates for the diphtheria/tetanus/pertussis vaccine (given on the same schedule as the 116E vaccine).[35] In each cycle, the number of individuals shedding rotavirus is totaled; then the daily probability of a fully susceptible individual contracting rotavirus, denoted *P*_*global*_, is given by

$$P_{global}=\left(\frac{T}{Mean\;duration\;of\;shedding}\right)\;\left(\frac{\#\;currently\;infected}{Total\;active\;population}\right)$$ (1)

where *T* is the transmission constant, representing the number of individuals in a fully susceptible population who would be infected by a single infectious case. The value of *T* was calibrated so that, in the absence of vaccination, the model produced cumulative incidence of rotavirus infection similar to that reported in the 2011 Indian cohort study by Gladstone et al[28]. Specifically, our calibration targets were the cumulative incidence of first infections at 6, 12, 24 and 36 months of age, and the cumulative incidence of second, third, and fourth infections at 36 months of age. Consistent with recent dynamic rotavirus models[29, 30], we assumed that second infections would be 50% as infectious as first infections and that subsequent infections would be 20% as infectious as first infections. **Fig 1** shows the cumulative incidence curves generated by the model with the calibrated *T* value of 5.1 plotted along with the calibration targets given by the Gladstone data.

**Fig 2:**
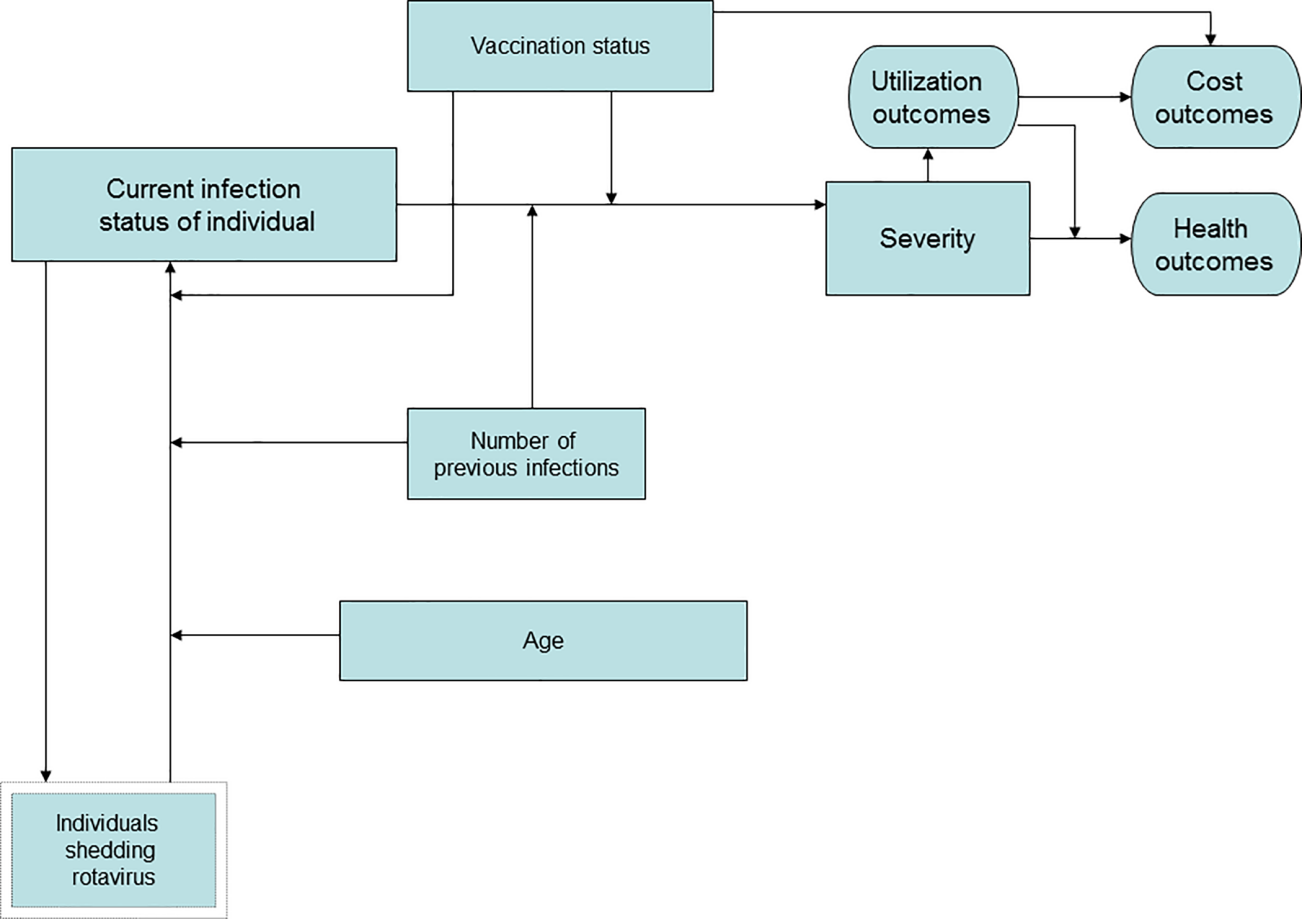
Model schematic. Factors (rectangles) which determine each individual’s probability of infection during a given model cycle and, ultimately, their outcomes (ovals) in terms of health services utilization, cost, and health. Single rectangles represent individual-level factors, and the double rectangle represents a population-level factor.

**Fig 2** describes the factors which determinue each individual’s probability of infection during a given model cycle and, ultimately, their outcomes in terms of health services utilization, cost, and health. Each cycle, for every active individual, an individualized probability of infection *P*_*i*_ is calculated by multiplying *P*_*global*_ by individualized relative risks based on age, natural infection history, and vaccination status.

$$P_{i}=P_{global}(RR_{age})(RR_{infection})(RR_{vaccination})$$ (2)

*RR*_*age*_ increases linearly from 0 to 1 between birth and thirteen weeks of age to account for declining levels of protective maternal rotavirus antibodies.[52] The relative risk explained by naturally-acquired immunity is given by

$$RR_{infection}=1-\gamma_{k}$$ (3)

where *γ*_*k*_ is the efficacy of *k* = 0, 1, 2, or 3 previous infections against any (including asymptomatic) future infection as reported by Gladstone.[28]

Relative risk of any rotavirus infection owing to vaccination effectiveness is given by

$$RR_{vaccination}=1-(p_{d})\delta_{any}$$ (4)

where *p*_*d*_ is the proportion of full efficacy conferred by *d* = 0, 1, 2, or 3 doses of the vaccine, and *δ*_*any*_ is the efficacy of the vaccine against any (including asymptomatic) infection. The value of *δ*_*any*_ is expressed as the projected India-specific vaccine efficacy against *severe* infection *δ*_*sev*_ as reported by Bhandari et al[15] adjusted by the ratio of efficacy of a single *natural* infection against any disease to that against severe disease *ζ* [28], that is,

$$\delta_{any}=\delta_{sev}(\zeta )$$ (5)

Although not shown here, we applied a waning factor for vaccine-induced protection of 8.6% per year after the first year of vaccination.[5, 8]

Once infected, a duration of infection (and thus viral shedding) is assigned randomly from a Poisson distribution with a mean of seven days.[25, 31] Regardless of the assigned duration of shedding, individuals in the model cease shedding when they die.

The probability of symptoms for those infected is calculated as

$$\alpha_{k}\;\left(1-I_{vac}[1-(\frac{1-\delta_{sx}}{1-\delta_{any}})]\right)$$ (6)

where *α*_*k*_ is the probability that infection *k* will be symptomatic given no vaccination, *δ*_*sx*_ is the efficacy of the vaccine against symptomatic infection, *δ*_*sx*_ > *δ*_*any*_, and *I*_*vac*_ is equal to 1 if an individual has had at least one dose of the vaccine, and 0 otherwise.

Further, among the symptomatic, the probability that symptoms will be severe (> = 11 on the Vesikari scale[34]) is calculated as

$$\beta_{k}\;\left(1-I_{vac}[1-(\frac{1-\delta_{sev}}{1-\delta_{sx}})]\right)$$ (7)

where *β*_*k*_ is the probability that infection *k*, if symptomatic, will be severe given no vaccination, *δ*_*sev*_ is the efficacy of the vaccine against severe infection, and *δ*_*sev*_ > *δ*_*sx*_.

Severity-dependent utilization probabilities were calculated as described in Rose et al[11] (Table 3). Symptomatic individuals could receive treatment either as an inpatient, an outpatient, or at home. In the last case, the individual would receive oral rehydration solution (ORS) with a probability corresponding to known ORS coverage rates.[41] Consistent with recent experience in India[39, 53], only those with severe symptoms who receive no formal (i.e. inpatient or outpatient) medical treatment were at risk for death. The probability of death for such individuals was calibrated so that mortality in the no vaccination arm matched known Indian rotavirus mortality of 1/242 by age five.[32]

For the first thirteen simulated weeks of the model, we extrinsically introduced the smallest possible number of cases each day that would lead to sustained, endemic infection over the full time horizon of the model. For purposes of comparing outcomes between the vaccination and no vaccination arms, we divided the model’s 30-year time horizon into four phases. First, a ten-year “run-in” phase with no vaccination allowed the building of a population with individuals of every possible age from zero to 1825 days (five years) and allowed an equilibrium pattern of rotavirus epidemics to establish itself prior to the introduction of vaccination. The second phase—“vaccine introduction”—consisted of the initial five years of vaccination. The following ten years constituted a third phase during which vaccination was fully implemented; this “full implementation” phase was the focus for our primary analysis. Phase four, the final five years, represented a “tail phase” since individuals born therein would not receive a full five years of follow-up. This phase was nonetheless necessary since such individuals played a role as reservoirs of rotavirus infection, and it allowed the individuals born at the end of phase three to experience a full five years of follow-up.

A microsimulation was chosen over a more traditional, deterministic compartmental model in order to accommodate the large number of potential risk profiles made possible by differing infection and vaccination histories and differing levels of symptoms.

### Outcome measures

We projected the vaccination-attributable reduction in rotavirus infections including asymptomatic infections, symptomatic episodes, severe episodes, and deaths, as well as the reduction in rotavirus-related utilization including home treatment with ORS, outpatient visits (emergency department and clinic combined), and hospitalizations. We also calculated the incremental cost-effectiveness ratio (ICER) for moving from a strategy of no rotavirus vaccination to a strategy of universal 116E vaccination. In the main analysis, the ICER was expressed in 2014 Indian rupees (Rs) per non-age-weighted disability-adjusted life year (DALY) averted and was calculated as the ratio of the mean net increase in direct medical costs under a program of vaccination to the mean DALYs averted as a result of such a program. For cost-effectiveness calculations, both rupees and DALYs were discounted at standard 3% annual rates.[47] As a conservative benchmark for cost-effectiveness, we used one times per-capita gross domestic product (pcGDP), a level considered “highly cost-effective” by the World Health Organization WHO.[54]

In a secondary analysis, we examined outcomes in the phase 2 vaccine introduction cohort (those born during the first five years of vaccination). To varying degrees, this group would not have received the full benefit of herd protection due to the coexistence of slightly older children who were born too late to benefit directly from the new vaccination program and who thus may have had higher levels of infection and shedding.

### Sensitivity analysis

We performed deterministic one-way sensitivity analyses on all non-calibrated parameters as well as multi-way sensitivity analysis of key sets of parameters. Ranges are shown in Tables 1–3.

## Results

**Table 4** shows the expected rotavirus-related clinical and utilization events per 100,000 Indian children under strategies of no vaccination and vaccination during the full implementation phase (beginning five years after initial implementation). Note that the gravest outcomes and the highest levels of utilization tended to be impacted most by vaccination. In fact, the number of rotavirus infections overall decreased by only 11.3%, while the number of severe infections decreased by 34.6%. Mean age at initial infection for children born in phase 3 was 236 days under a strategy of no vaccination and 311 days under s strategy of vaccination. We found little difference in the projected mortality reduction from vaccination for children born in the vaccine introduction phase versus those born in the full implementation phase (34.4% versus 34.6%).

**Table 4:** Expected clinical events and utilization in a birth cohort of 100,000 Indian infants followed for 5 years under strategies of *no vaccination* and *vaccination* using 116E.

	No vaccination	Vaccination	Change (%)
Rotavirus-related clinical events in a cohort of 100,000 children followed for 5 years			
Any infection	345,953	306,809	-11.3
Asymptomatic infections	238,597	213,402	-10.6
Symptomatic infections (including severe)	107,356	93,407	-13.0
Severe infections	20,584	13,472	-34.6
Death	410	268	-34.6
Rotavirus-related utilization in a cohort of 100,000 children followed for 5 years			
Hospitalization	2,638	1,897	-28.1
Outpatient visits	24,110	18,976	-21.3
Home treatment using ORS	21,022	18,945	-9.9

Full implementation phase cost-effectiveness results are displayed in **Table 5**. The table shows mean direct medical costs per child from an all-payer perspective under strategies of no vaccination and vaccination. The marginal cost represents the increased cost of moving from the former strategy to the latter. Similarly, DALYs are compared and the difference expressed as the mean number of DALYs averted. The model predicts that a fully implemented vaccination program would avert 0.0404 DALYs per child at a marginal cost of Rs 138 (approximately $2.27) per child. The resulting incremental cost-effectiveness ratio (ICER) of Rs 3,429 per DALY averted (approximately $56) falls well below the WHO criterion for “very cost-effective” interventions of one times pcGDP (current Indian pcGDP is approximately Rs 88,500 [55]). The program is slightly more cost-effective from the societal perspective at an ICER of Rs 3,384 as the additional cost savings to families in terms of transportation costs and lost productivity are incorporated. Expressed in terms of cost per age-weighted DALY averted, the all-payer-perspective ICER was Rs 2,991 per DALY averted. Without adjustment for disability, vaccination cost an estimated Rs 3,464 per life year saved. Finally, at a net cost of 138.4 Rupees per infant born in India, and with an annual birth cohort of approximately 25 million[56], universal 116E vaccination would cost around Rs 3.48 billion ($57 million) annually net of treatment savings.

**Table 5:** Incremental cost-effectiveness of moving to a strategy of universal 116E vaccination. (ICER = incremental cost-effectiveness ratio); values are *per child*.

	Mean cost (2014 Rupees)	Marginal cost	Mean DALYs	DALYs Averted	ICER (Rs / DALY averted)
No vaccination	139.0	—	1.6399	—	
Vaccination	277.4	138.4	1.5995	0.0404	**3429**

### Sensitivity analysis

**Fig 3:**
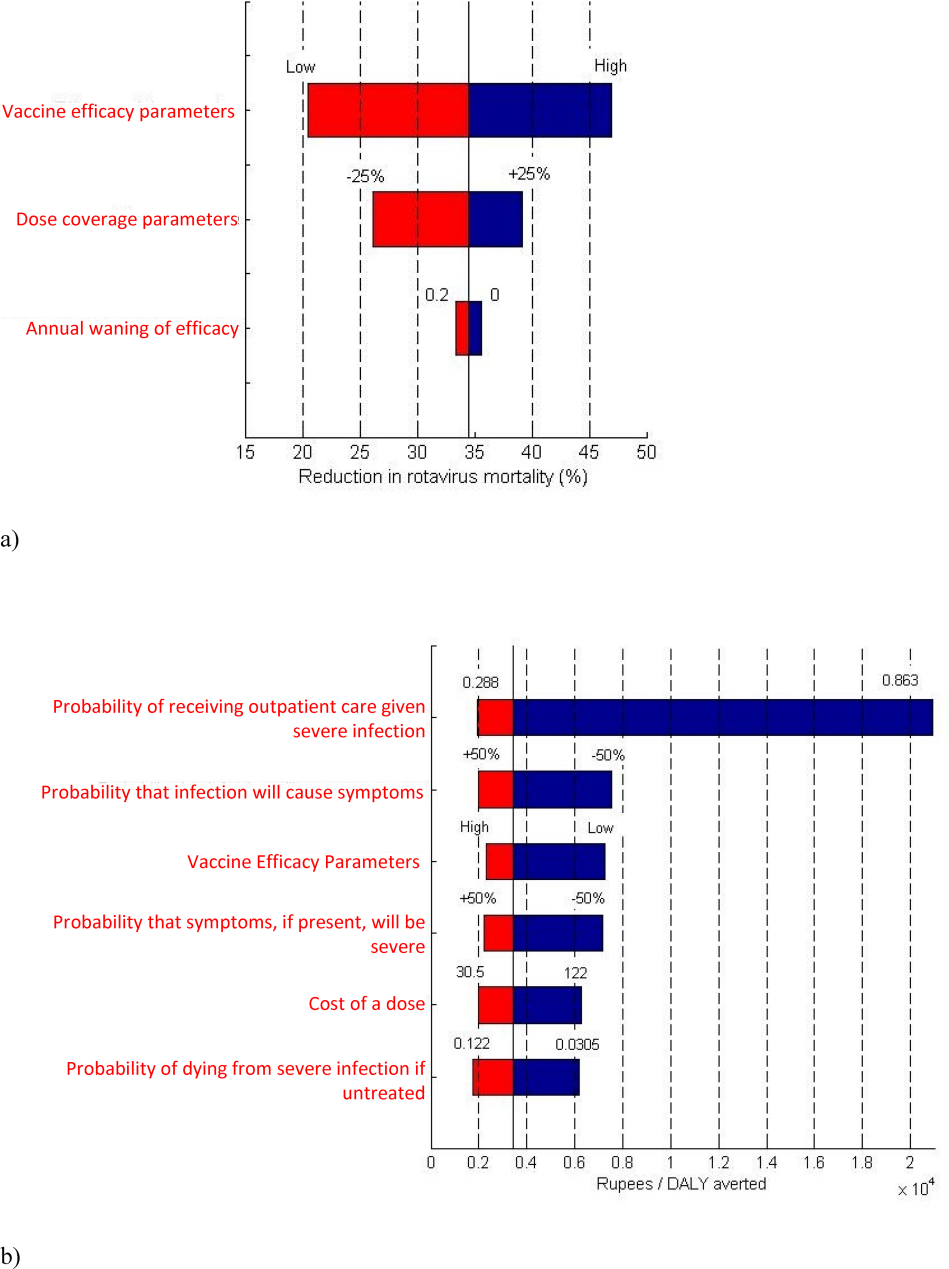
Highlighted sensitivity analysis results. a) Model parameters which, when varied over the ranges specified in Tables 1–3, have the greatest impact on rotavirus mortality reduction. The percent reduction in rotavirus mortality is shown on the x-axis. The solid black line indicates the baseline mortality reduction (34.6%). Bounds expressed in percentages (e.g. +/-25%), represent relative percentages. b) Model parameters which, when varied, have the greatest impact on the ICER. The ICER (in Rs) is on the x-axis. The solid black line indicates the baseline ICER (Rs 3,429 per DALY averted).

Rotavirus mortality was most sensitive to changes in vaccine efficacy. If vaccine efficacy against asymptomatic, symptomatic, and severe rotavirus were simultaneously reduced to their lower bounds (based on 95% confidence intervals from the 2014 116E vaccine trial[15]), rotavirus mortality reduction would fall from 34.6% to 20.5% with an ICER of Rs 7,238per DALY averted. To a lesser extent, vaccination levels were consequential for mortality. If vaccine coverage increased to 100% for doses one and two, and 90% for dose three (equivalent to coverage levels seen in the top quartile of Indian states [57]), then mortality reduction would rise from 34.6% to 39.0%. **Fig 3A** depicts the model parameters which, when varied to the extremes of their ranges, had the most impact on mortality, while **Fig 3B** depicts the parameters which most impacted cost-effectiveness. Cost-effectiveness was most sensitive to changes in the probability that those with severe symptoms would receive outpatient care. When this probability was increased by 50% (from 0.575 to 0.863), the cost per DALY averted increased to Rs 20,910. Underlying this shift was a roughly 86% decrease in mortality owing to improved treatment access that was independent of vaccination. Though not as cost-effective in this scenario, the larger ICER was equivalent to only about ¼ of the one times pcGDP criterion.

## Discussion

Our analysis suggests that a policy of universal 116E vaccination of Indian infants would reduce rotavirus-related mortality in that country by 34.6%, corresponding to over 34,000 lives saved annually despite relatively modest vaccine efficacy. In addition, rotavirus-related hospitalization would be expected to decrease by 28.1%, and rotavirus-related outpatient visits by 21.3%. Incorporating treatment savings, we predict that an established vaccination program would cost Rs 3,429 (approximately $56) per DALY averted, a small fraction of the 2014 Indian per capita GDP of Rs 88,500, a typical criterion for “highly cost-effective” interventions. Conclusions of the model were stable across a wide range of assumptions including very conservative lower estimates of vaccine efficacy. In addition to cost-effectiveness, it is crucial to examine total program costs when considering such a large-scale public health endeavor in a resource-limited setting. We estimate that universal 116E vaccination in India would cost around $57 million annually, or 0.2% of the Indian Government’s 2014 spending on health care.[58] Though there are opportunity costs associated with spending on any program, this level of financial burden would seem sustainable, especially given its life-saving potential.

One set of investigators recently examined a hypothetical program of universal vaccination in India using 116E[18], and two groups have produced analyses involving an unspecified vaccine with characteristics similar to 116E.[19, 20] Compared to these analyses, the current work is based on a more realistic dynamic disease transmission model which accounts for the immunizing effect of asymptomatic infections and for indirect effects of vaccination that accrue to the unvaccinated. In terms of estimated disease burden reduction, however, our model’s results were similar to those of the other investigators. John et al[18] modeled outcomes of introducing the 116E vaccine in India based on five cohorts of children receiving free access to medical care. They estimated a 34.3% reduction in mortality, a 33.4% reduction in hospitalizations, and a 21.0% reduction in outpatient visits. They did not report cost-effectiveness. Megiddo et al [19] used an agent-based simulation model to predict outcomes of implementing an unspecified rotavirus vaccine with efficacy similar to that of the 116E vaccine. At coverage levels equivalent to current DTP coverage, they estimated a 34.7% reduction in mortality with an incremental cost-effectiveness of $71 per non-age-weighted DALY averted (compared to $56 from our model). These cost-effectiveness results did not incorporate treatment savings or out-of-pocket expenditures, however. Estimates of decreased morbidity, hospitalization, or outpatient utilization were not reported. Finally, Rheingans et al [20] predicted a 33.7% reduction in mortality at a cost of $118 per age-weighted DALY averted (compared to Rs 2,991 or $49 from our model). Rheingans assumed a vaccination cost of $1.25 per dose plus a $1.25 non-vaccine cost per dose, compared to the inputs of $1.00 and $0.56 for vaccine procurement and non-vaccine costs, respectively, used in our model. $1.25 represents a fairly high non-vaccine cost estimate.[59] When both inputs were changed to $1.25 in our model, the ICER rose to a value equivalent to $85 per age-weighted DALY averted.

Note also that there was little difference in the population effect of vaccination for children born in phase 2 (five year vaccine introduction phase) of our model versus those born in phase 3 (full implementation). During phase 2, a number of children active in the model were born prior to implementation of vaccination (i.e. born prior to vaccine introduction but alive and less than five years old during the vaccine introduction phase). As such, children born during the vaccine introduction phase would likely be exposed to greater levels of infection from slightly older children in their environments than would their counterparts born during the full implementation phase, during which all children under five had a chance to receive vaccination. The fact that the impact of vaccination was nearly identical for children born in these two phases suggests that the indirect benefit of vaccination among those who were eligible for vaccination but did not receive it was minimal. This is true despite also accounting for the role of asymptomatic infections in transmission. The same conclusion is suggested by the previously noted similarity between our results and those from recent models that did not account for indirect effects.[18, 20] An explanation for this lack of significant indirect benefit may be that while slightly less circulating infection lowers the daily risk of infection for the unvaccinated, virtually everyone will eventually contract a first infection at some point before age five. Those who are not vaccinated, though, do not receive the severity-attenuating benefits of vaccination, so the net effect is that infection is *delayed* somewhat, but morbidity, mortality, and utilization are affected little for unvaccinated children.

A direct comparison of efficacies in the Indian setting between the 116E vaccine and the western-made monovalent and pentavalent vaccines is not possible given available data. We can, however, compare 116E efficacy data from India with pooled efficacy estimates of these vaccines in other high mortality countries. One-year efficacy against severe disease for 116E in high mortality settings was 56.4% (95% CI 36.6% to 70.1%)[15] compared with 63% (95% CI 25% to 82%) and 57% (95% CI 38% to 71%) for the western-made monovalent and pentavalent vaccines, respectively.[60] However, the two-year 116E efficacy of 53.6% (95% CI 35.0 to 66.9)[15] is higher than the analogous figures for the other vaccines: 42% [95% CI 21% to 58%] and 41% [95% CI 18% to 57%] for RotaRix and RotaTeq, respectively.[59] Less similar are the expected price differentials between 116E and the western vaccines. While Bharat Biotechnic, manufacturer of the 116E vaccine, has pledged to offer the vaccine at a cost of less than $1 per dose (less than $3 per course) [15], vaccine costs for RotaRix and RotaTeq are expected to be $5 and $10.50 per course, respectively.[9] India has emerged as a major manufacturer of relatively inexpensive vaccines for the developing world in recent decades. With increasingly sophisticated capabilities, the Indian vaccine industry is poised to help make affordable delivery of more difficult-to-manufacture vaccines such as the rotavirus vaccine a reality.[61]

Finally, it is worth mentioning that improvement in access to outpatient care for the severely ill of less than 30 percentage points in sensitivity analysis resulted in an 86% reduction in rotavirus mortality even without vaccination. This result hints at the substantial benefit that health system improvements might yield in terms of mortality reduction from rotavirus as well as from numerous other causes of pediatric death in India.

### Limitations

Though our main source for treatment cost estimates was comprehensive in that it included non-medical and indirect costs needed to assess net program costs from a societal perspective, these data were only gathered from hospitals and clinics within one town in India.[45] All cost inputs were varied extensively in sensitivity analysis. It should be noted that the only cost input to which cost-effectiveness results showed any substantial degree of sensitivity was vaccine cost (Fig 3B). Even with a doubling of vaccine cost, however, vaccination remained very cost-effective. Also, the only comparator in our model for a universal rotavirus vaccination program was the scenario of no universal vaccination. A number of alternative comparisons could be made with competing potential uses of the same funds. In order to facilitate these comparisons by decision makers, we included baseline cost-effectiveness results expressed in the commonly used unit of cost per DALY averted. Third, we did not explicitly model different regions or subpopulations within India. Undoubtedly, a program of rotavirus vaccination would benefit some groups more than others, or would cost more in some groups than in others. Our approach estimated nationwide average outcomes.

We chose to model only individuals under five. The rationale for this decision was two-part: 1) a lack of reliable parameter estimates to describe factors in older individuals such as shedding duration, efficacy of *n*th natural infection (where *n* is high), and extended rates of vaccine efficacy waning; and 2) the extreme rarity of mortality in older age groups. While the model is calibrated to achieve rotavirus incidence and mortality matching empirical data for the age group of interest, exclusion of older groups from the model may overstate the impact of vaccination on transmission to the extent that older individuals act as reservoirs.

## Conclusion

Our results suggest that vaccination of Indian infants with 116E would save over 34,000 lives annually at a cost per DALY averted which would be considered highly cost-effective by WHO standards, and with a net program cost representing only a small fraction of Indian healthcare expenditures. Estimates of mortality reduction are similar to those from two recent analyses of 116E vaccination in India. It appears that, given a sufficiently long timeframe of analysis, inclusion of herd immunity effects is not necessary for accurately estimating the mortality impact of rotavirus vaccination since that impact appears to come primarily from severity attenuation for vaccinated individuals.
